# Accumulation of advanced glycation end products (AGEs) is associated with the severity of aortic stenosis in patients with concomitant type 2 diabetes

**DOI:** 10.1186/s12933-020-01068-7

**Published:** 2020-06-17

**Authors:** Magdalena Kopytek, Michał Ząbczyk, Piotr Mazur, Anetta Undas, Joanna Natorska

**Affiliations:** 1 John Paul II Hospital, Kraków, Poland; 2 Institute of Cardiology, Jagiellonian University Medical College, 80 Pradnicka St, 31-202 Kraków, Poland

**Keywords:** Aortic stenosis (AS), Diabetes mellitus (DM), Advanced glycation end products (AGEs), Inflammation, Oxidative stress

## Abstract

**Background:**

Accumulation of advanced glycation end products (AGEs) leads to chronic glycation of proteins and tissue damage, particularly in patients with diabetes mellitus (DM). We aimed to evaluate whether increased accumulation of AGEs in patients with aortic stenosis (AS) and concomitant type 2 diabetes (DM) is associated with AS severity.

**Methods:**

We prospectively enrolled 76 patients with severe AS (47.1% males; nonDM), aged 68 [66–72] years, and 50 age-matched DM patients with a median blood glucose level of 7.5 [5.9–9.1] mM and glycated hemoglobin (HbA1c) of 6.8 [6.3–7.8]%, scheduled for aortic valve replacement. Valvular expression of AGEs, AGEs receptor (RAGE), interleukin-6 (IL-6), and reactive oxygen species (ROS) induction were evaluated ex vivo by immunostaining and calculated as the extent of positive immunoreactive areas/total sample area. Plasma levels of AGEs and soluble RAGE (sRAGE) were assessed by ELISAs.

**Results:**

Subjects with DM had increased valvular expression of both AGEs (6.6-fold higher, 15.53 [9.96–23.28]%) and RAGE (1.8-fold higher, 6.8 [4.9–8.45]%) compared to nonDM patients (2.05 [1.21–2.58]% and 2.4 [1.56–3.02]%, respectively; both p < 0.001). Plasma levels of AGEs (12-fold higher) and sRAGE (1.3-fold higher) were elevated in DM patients, compared to nonDM (both p < 0.0001). The percentage of valvular ROS-positive (2.28 [1.6–3.09] vs. 1.15 [0.94–1.4]%, p < 0.0001) but not IL-6-positive areas was higher within DM, compared to nonDM valves. In DM patients, the percentage of valvular AGEs- and RAGE-positive areas correlated with HbA1c (r = 0.77, p < 0.0001 and r = 0.30, p = 0.034). Similarly, plasma AGEs and sRAGE levels were associated with HbA1c in the DM group (r = 0.32, p = 0.024 and r = 0.33, p = 0.014, respectively). In all DM patients, we found an association between the amount of valvular AGEs and the disease severity measured as aortic valve area (AVA; r = 0.68, p < 0.0001). Additionally, in DM patients with HbA1c > 7% (n = 24, 48%) we found that valvular expression of AGEs correlated with mean transvalvular pressure gradient (PG_mean_; r = 0.45, p = 0.027). Plasma AGEs levels in the whole DM group correlated with AVA (r = − 0.32, p = 0.02), PG_mean_ (r = 0.31, p = 0.023), and PG_max_ (r = 0.30, p = 0.03).

**Conclusions:**

Our study suggests that poorly-controlled diabetes leads to increased AGEs and RAGE valvular accumulation, which at least partially, might result in AS progression in DM patients.

## Background

Aortic stenosis (AS) is the most common cause of acquired valvular heart disease in the adult population, with 2–3% prevalence for individuals older than 65 years in developed countries [1]. AS is initiated as aortic valve sclerosis, associated with a mild valve thickening. Histopathologic heterogeneity of AS indicates the involvement of cell dependent mechanisms that regulate calcium load in the valve leaflets, as well as the participation of different cell types, including valvular interstitial cells (VICs), endothelial cells and cardiac leukocytes [2]. Under pathological conditions, such as inflammation or oxidative stress VICs can differentiate into myofibroblasts (causing fibrosis) and osteoblast-like cells (causing calcification) [2]. Diabetes mellitus (DM), a known cardiovascular risk factor, has also been reported as a risk factor for AS progression [3]. In patients with AS and concomitant type 2 DM reduced systemic arterial compliance and left ventricular dysfunction at the midwall level, corresponding to slightly depressed myocardial contractility have been shown [4]. Chronic hyperglycemia is a common feature of DM and has been related to vascular and inflammatory cell interactions with advanced glycation end products (AGEs) [5]. Advanced glycation is the irreversible attachment of reducing sugars to the free amino groups of proteins. Conditions such as DM rapidly accelerate AGE formation [6]. It has been shown that AGEs accumulation plays a key role in development of vascular calcification in DM patients [7–9]. AGEs, which are a heterogeneous group of molecules generated through non-enzymatic glycation and oxidation of proteins such as collagen, elastin and laminin [10–12], lipids, and nucleic acids [10] can alter tissue function and its mechanical properties [10–12]. Interestingly, it has been shown that chronic high dietary AGEs lead to increased arterial stiffness with subsequent elevation of systolic blood pressure and inflammatory activation, which may lead to the development of vascular complications in type 2 DM [11]. AGEs modulate multiple cellular processes through the cross-linking of intracellular and extracellular matrix proteins [12] or binding to their cell surface receptor (RAGE), which produces excesses in inflammatory molecule production [13, 14]. It has been shown that elevated circulating soluble RAGE (sRAGE) levels are associated with the presence of bicuspid aortic valve and associated aortopathies, independently of aortic diameter [15]. Moreover, Hofmann et al. [16] have shown in model using RAGE knock-out mice that both AGEs and RAGE are involved in the aortic leaflets calcification and subsequent AS. Low levels of both sRAGE and endogenous secretory receptor for RAGE (esRAGE), which are considered to be protective against AGEs, were shown as a very early marker of initial target organ damage in mild hypertensives [17] or a factor associated with negative coronary artery remodeling in diabetics [18]. Moreover, the concentration of sRAGE has been associated with increased arterial wall stiffening over time [19]. Despite numerous reports on AGE and RAGE involvement in the pathogenesis of cardiovascular diseases little is known about mechanisms by which hyperglycemia-related increase of AGEs and RAGE affects inflammation and calcification within aortic stenotic valves. We aimed to investigate whether in AS patients with concomitant DM, the severity of AS is associated with enhanced accumulation of both valvular and plasma AGEs and RAGE.

## Methods

### Study subjects

We enrolled 126 patients with isolated AS on tricuspid aortic valve (nonDM group), including 50 AS patients with concomitant type 2 DM (DM group). Patients were hospitalized between August 2016 and February 2019 in the Department of Cardiovascular Surgery and Transplantology at the John Paul II Hospital, Krakow, Poland. Severe AS was defined by a mean transvalvular gradient (PG_mean_) ≥ 40 mmHg and/or aortic valve area (AVA) < 1 cm^2^, based on transthoracic echocardiography, performed by an experienced cardiologist on a Toshiba APLIO 80 (Toshiba, Tokyo, Japan) ultrasound machine. All patients were scheduled to undergo aortic valve replacement, following the Heart Team indication. Left ventricular ejection fraction (LVEF) was routinely measured during echocardiography. The diagnosis of atherosclerosis was based on angiographically documented coronary artery stenosis > 20% diameter. Patients classified as having DM had to be diagnosed preoperatively based on fasting plasma glucose ≥ 7.0 mmol/L (126 mg/dL) on two separate occasions, HbA1c equal or exceeding 6.5% (48 mmol/mol) or oral glucose tolerance test of 140 mg/dL (7.8 mmol/L) to 199 mg/dL (11.0 mmol/L) according to The International Expert Committee [20] and only individuals receiving insulin or oral hypoglycemic agents preoperatively were included in this study. Hypercholesterolemia was diagnosed based on medical records, cholesterol-lowering therapy or total cholesterol of 5.0 mmol/L or more. Arterial hypertension was diagnosed based on a history of hypertension (blood pressure > 140/90 mmHg) or preadmission antihypertensive treatment. Smoking was defined as the use of one or more cigarettes per day. The exclusion criteria were: angiographically documented coronary artery stenosis > 20% diameter, rheumatic AS, acute infection, diagnosed malignancy, endocarditis, chronic kidney disease, any previous heart surgeries, acute cardiovascular event in the last 3 months before inclusion, any concomitant valvular surgery and pregnancy. The valvular anatomy was confirmed intraoperatively by a cardiac surgeon, and bicuspid valve was used as an exclusion criterion. The Local Ethical Committee in Krakow approved the study and participants provided informed consent in accordance with the Declaration of Helsinki.

### Laboratory analysis

At the day of valve replacement, after an overnight fast venous blood was drawn between 7:00 and 9:00 AM from the antecubital vein into citrated (9:1 of 0.106 M sodium citrate) or EDTA (both SARSTEDT, Nümbrecht, Germany) tubes. Blood was centrifuged at 2500*g* at 20 °C for 20 min and stored in aliquots at − 80 °C until analysis. Blood drawn into serum tubes was centrifuged at 1600*g* at 4 °C for 10 min and stored at − 80 °C. To determine lipid profile, glucose and creatinine in serum, routine laboratory assays were used. Serum high-sensitivity C-reactive protein (hsCRP) was determined using immunoturbidimetry (Roche Diagnostics, Mannheim, Germany). Fibrinogen was measured by the von Clauss method in citrated plasma (Instrumentation Laboratory, Bedford, MA, USA). Glycated hemoglobin HbA1c was determined using turbidimetric inhibition immunoassay TINIA in hemolysate prepared from whole blood (Roche Diagnostics, Mannheim, Germany). Serum fructosamine levels were measured using a colorimetric assay (Roche Diagnostics, Risch-Rotkreuz, Switzerland).

### Enzyme-linked immunosorbent assay (ELISA)

Human advanced glycation end products (AGEs; all species with the predominance of N(6)-Carboxymethyllysine) and circulating soluble human receptor for advanced glycation end products (sRAGE) concentrations in EDTA plasma samples were assayed quantitatively using commercial ELISA kits (EIAab, Wuhan, China, no. E0263h and Boster Biological Technology, California, USA, no. EK0827, respectively) in accordance with manufacturer’s instructions. Serum insulin levels were assessed using Insulin Human ELISA Kit (Invitrogen —Thermo Fisher Scientific, Waltham, MA, USA).

### Aortic valves preparation

Valve samples embedded in Tissue Tec-OCT compound (Sakura, Torrance, CA, USA) were cryosectioned (4.5 μm thick sections) onto SuperFrost slides (Menzel-Glaser, Braunschweig, Germany) by a Leica CM 1520 cryostat. Transverse sections were taken from the mid and commissural areas of the leaflet and stored at − 20 °C until immunostaining.

### Immunofluorescence analysis

Sections were fixed in ice-cold methanol-acetone (1:1) mixture. After endogenous peroxidase activity quenching with 3% H_2_O_2_ (15 min) at room temperature (RT) and blocking of unspecific background with 3% bovine albumin (BSA, Sigma Co, St. Louis, MO, USA) at RT in dark (30 min). Primary adequate antibodies against AGE (1:500; no. ab23722), tissue RAGE (1:100; no. ab3611), interleukin-6 (IL-6, 1:800; no. ab6672), and reactive oxygen species (ROS) modulator 1 (ROMO1, indirectly evaluating ROS production; 1:100; no. ab121379) (all from Abcam, Cambridge, UK) were applied overnight at 4 °C. Primary antibodies were followed by the corresponding secondary goat or mouse antibodies conjugated with fluorochrome AlexaFluor 488 or AlexaFluor 594 (Abcam, Cambridge, UK) (1:1000) at 4 °C for 1 h in dark. Double-label immunofluorescence analyses were performed using the same antibodies. The co-localization of AGE and RAGE was performed. A negative control (without primary antibody incubation) was included routinely to all staining. Images were analyzed using Olympus BX 43 microscope equipped with software (Cell Sense Standard, version 11.0.06). Per each valve 30 images were taken and the percentage of positively-stained areas was calculated as the extent of positive immunoreactive areas/total sample area as follows [21]:

$$\frac{\varSigma \;of\; immunoreactive\;areas}{Total\;section\;area}$$

The fluorescence intensity (FI) was computed as the ratio of fluorescence of positively and negatively stained areas. The investigators analyzing the data were blinded to the study groups. The ratio of valvular AGEs and sRAGE (AGEs/sRAGE) has also been calculated [22].

### Statistical analysis

All statistical analysis were performed using STATISTICA Version 12.5 (StatSoft STATISTICA™, Poland) program. Categorical variables were presented as numbers and percentages and were analyzed by Pearson’s χ^2^ or two-tailed Fisher’s exact test. Continuous variables were expressed as mean ± standard deviation (SD) or median and interquartile range [IQR]. Normality was analyzed by the Shapiro–Wilk test. The Student’s test was used for normally distributed continuous variables. Differences between groups were compared using the Mann–Whitney U test for non-normally distributed continuous variables. Associations between nonparametric variables were assessed by Spearman’s tests. The multivariate analyses were performed using linear regression models adjusted for potential confounders such as age, hypertension, hypercholesterolemia, and the use of statin and/or angiotensin-converting enzyme inhibitors. P-values of < 0.05 were considered as statistically significant. The study was powered to have a 90% chance of detecting a 15% difference in valvular expression of different factors using a p-value of 0.01, based on the previous study [14]. In order to demonstrate such a difference or grater, the group size is equal to 44 patients, or more is required in each group.

## Results

DM patients did not differ from the nonDM group with regard to demographic factors, but were more frequently obese (BMI > 30 kg/m^2^, Table 1). More DM patients used statins, beta-blockers and angiotensin converting enzyme (ACE) inhibitors compared to subjects from the nonDM group (all p < 0.05; Table 1). The median time of DM duration was 11 [7–18] years, and DM patients had 44.2% higher glucose, 25.9% higher HbA1c, and 16.4% higher fructosamine levels compared to nonDM subjects (all p < 0.05; Table 1). DM patients were also characterized by slightly poorer renal function (Table 1). No intergroup differences were found in other laboratory parameters (Table 1). Among DM patients, as many as 24 (48%) individuals had poorly controlled diabetes, defined as HbA1c > 7% (Table 2).

**Table 1:** Characteristics of patients with aortic stenosis with or without concomitant diabetes mellitus (DM)

Variable	NonDM patients (n = 76)	DM patients (n = 50)	p-value
Age, years	68 [66–72]	70 [66–74]	0.18
Male, n (%)	41 (53.9)	31 (62)	0.37
BMI, kg/m^2^	28.4 [26.6–31.2]	31.3 [28.7–34.5]	< 0.001
Risk factors, n (%)			
Arterial hypertension	70 (92.1)	50 (100)	0.08
Hypercholesterolemia	20 (26.3)	10 (20)	0.52
Current smoking	9 (11.8)	8 (16)	0.60
Medications, n (%)			
Beta-blockers	61 (80.3)	47 (94)	0.038
Acetylsalicylic acid	52 (68.4)	40 (80)	0.22
ACE inhibitors	53 (69.7)	45 (90)	0.008
Statins	56 (73.7)	46 (92)	0.011
Insulin	0	14 (28)	< 0.0001
Metformin	0	36 (72)	< 0.0001
Echocardiographic parameters			
Mean gradient, mmHg	48 [42–59]	52 [43–66]	0.067
Maximum gradient, mmHg	82.3 ± 14.2	89.2 ± 12.3	0.054
LVEF, %	60 [55–65]	60 [58–64]	0.39
AVA, cm^2^	0.86 [0.7–0.95]	0.78 [0.6–0.8]	0.048
Laboratory investigation			
Fibrinogen, g/L	3.4 ± 0.8	3.6 ± 0.6	0.28
Creatinine, µmol/L	74 [65–89]	81 [74–100]	0.011
hsCRP, mg/L	1.2 [1.0–4.0]	1.0 [1.0–2.0]	0.34
Glucose, mmol/L	5.2 [4.9–5.6]	7.5 [5.9–9.1]	< 0.0001
HbA1c, %	5.4 [5.2–5.7]	6.8 [6.3–7.8]	< 0.0001
Insulin, µIU/mL	16.5 [13.1–19.9]	16.4 [12.3–23.4]	0.41
Fructosamine, µmol/L	225 [217–236]	262 [241–291]	< 0.0001
TC, mmol/L	4.1 [3.7–4.8]	3.8 [3.0–4.6]	0.06
LDL-C, mmol/L	2.6 [2.1–3.3]	2.3 [1.5–3.1]	0.17
HDL-C, mmol/L	1.3 [1.1–1.6]	1.2 [1.0–1.4]	0.039
Triglycerides, mmol/L	1.2 [0.9–1.8]	1.5 [1.2–2.0]	0.27

Data presented as numbers (percentages), mean ± SD or medians [interquartile range]. Categorical variables were analyzed by the Chi square test. The Mann–Whitney U or Student tests were used to compare differences between groups

*AS* aortic stenosis, *DM* type 2 diabetes mellitus, *BMI* body mass index, *ACE inhibitors* angiotensin converting enzyme inhibitors, *LVEF* left ventricular ejection fraction, *AVA* aortic valve area, *hsCRP* high-sensitivity C-reactive protein, *HbA1c* glycated hemoglobin, *TC* total cholesterol, *LDL-C* low density lipoprotein cholesterol, *HDL-C* high density lipoprotein cholesterol

**Table 2:** Comparison of AS patients with concomitant type 2 DM stratified according to glycated hemoglobin levels (HbA1c)

Variable	Patients with DM and HbA1c > 7% (n = 24)	DM and HbA1c ≤ 7% (n = 26)	p-value
Age, years	74 [70–76]	70 [66–73]	0.12
Male, n (%)	13 (54.2)	18 (69.3)	0.38
BMI, kg/m^2^	30.4 [26.6–31.2]	32.2 [28.7–34.5]	0.30
Risk factors, n (%)			
Arterial hypertension	24 (100)	26 (100)	0.99
Hypercholesterolaemia	7 (29.2)	3 (11.5)	0.16
Current smoking	2 (8.3)	6 (23.1)	0.25
Medications, n (%)			
Beta-blockers	21 (87.5)	26 (100)	0.10
Acetylsalicylic acid	19 (79.2)	21 (80.8)	0.99
ACE inhibitors	22 (91.7)	23 (88.5)	0.99
Statins	21 (87.5)	25 (96.2)	0.34
Insulin	14 (58.3)	0	< 0.0001
Metformin	10 (41.7)	26 (100)	< 0.0001
Echocardiographic parameters			
Mean gradient, mmHg	61 [46–67]	44 [41–50]	0.003
Maximum gradient, mmHg	95 [72–109]	64 [58–77]	<0.00001
LVEF, %	60 [55–65]	60 [58–64]	0.98
AVA, cm^2^	0.65 [0.56–0.80]	0.85 [0.8–0.9]	< 0.0001
Laboratory investigation			
Creatinine, µmol/L	91 [88–101]	79 [73–99]	0.19
hsCRP, mg/L	1.0 [1.0–2.0]	1.0 [1.0–2.0]	0.53
Glucose, mmol/L	6.8 [6.2–8.4]	6.4 [5.5–7.5]	0.21
Insulin, µIU/mL	16.2 [12.4–23.3]	16.3 [12.4–23.4]	0.89
Fructosamine, µmol/L	271 [250–304]	259 [231–266]	0.04
Valvular AGEs, %	23.3 [17.5–28.1]	10.8 [9.3–13.5]	< 0.00001
Valvular RAGE, %	8.1 [6.6–8.5]	5.7 [4.8–7.7]	0.015
Plasma AGEs, ng/mL	9.3 [8.5–12.0]	9.6 [8.6–10.4]	0.55
Plasma sRAGE, pg/mL	1977 [1596–2455]	1988 [1517–2613]	0.70

Data presented as numbers (percentages), mean ± SD or medians [interquartile range]. Categorical variables were analyzed by the Chi square test. The Mann–Whitney U or Student tests were used to compare differences between groups

*AGEs* advanced glycation end products, *RAGE* receptor for AGEs, for abbreviations see Table 1

### Valvular expression of AGEs and RAGE

**Fig. 1:**
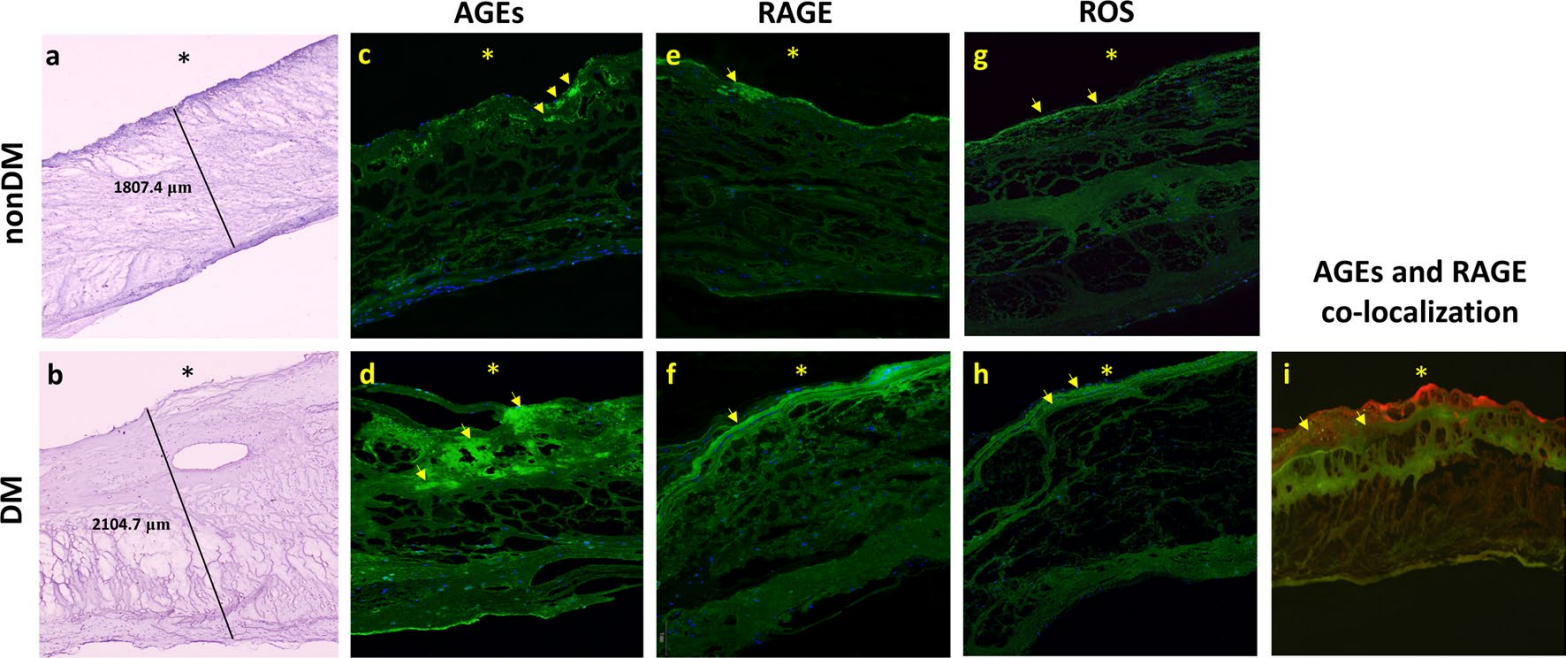
Expression of AGEs and RAGE within aortic stenotic valves. Representative microphotographs of aortic stenotic valves dissected from patients with aortic stenosis (nonDM) or aortic stenosis with concomitant type 2 diabetes mellitus (DM). **a**, **b** The thickness of the leaflets stained with hematoxylin, **c**, **d** the valvular expression of advanced glycation end products (AGEs) and **e**, **f** their receptor (RAGE), **g**, **h** the expression of reactive oxygen species (ROS) modulator 1 (indirect staining for ROS), and **i** co-localization of AGEs (green) and RAGE (red). Cell nuclei are stained in blue (DAPI). *Aortic site of the leaflet. Original magnification ×40

The thickness of the aortic valve leaflets was greater in DM patients compared to nonDM valves (1926.32 ± 197.85 µm vs. 1298.32 ± 183.37 µm, p = 0.017) (Fig. 1a, b).

Valvular expression of AGEs and RAGE was mostly detected at the aortic site of the leaflets and presented a diffused pattern of fluorescence for AGEs, whereas RAGE expression was more condensed and located mainly on the edge of the leaflets (Fig. 1c–f). Valvular AGE expression was 6.6-fold higher in DM compared to nonDM patients (15.53 [9.96–23.28]% vs. 2.05 [1.21–2.85]%, p < 0.001) (Fig. 1c, d). Similarly, in the DM group we found 1.8-fold higher expression of valvular RAGE than in the nonDM group (6.8 [4.9–8.45]% vs. 2.4 [1.56–3.02]%, p < 0.001) (Fig. 1e, f). Of note, AGEs and RAGE showed co-expression in 51.5% of positively stained areas (Fig. 1i). DM compared to nonDM valves were characterized by higher ROS-positive area (2.28 [1.6–3.09]% vs 1.15 [0.94–1.4]%; p < 0.0001; Fig. 1g, h). The valvular expression of IL-6 tended to be increased in DM compared to nonDM patients (1.57 [0.96–2.15]% vs. 1.18 [0.96–1.8]%; p = 0.062). There were no associations between valvular ROS or IL-6 expression and echo parameters in both patient groups.

**Fig. 2:**
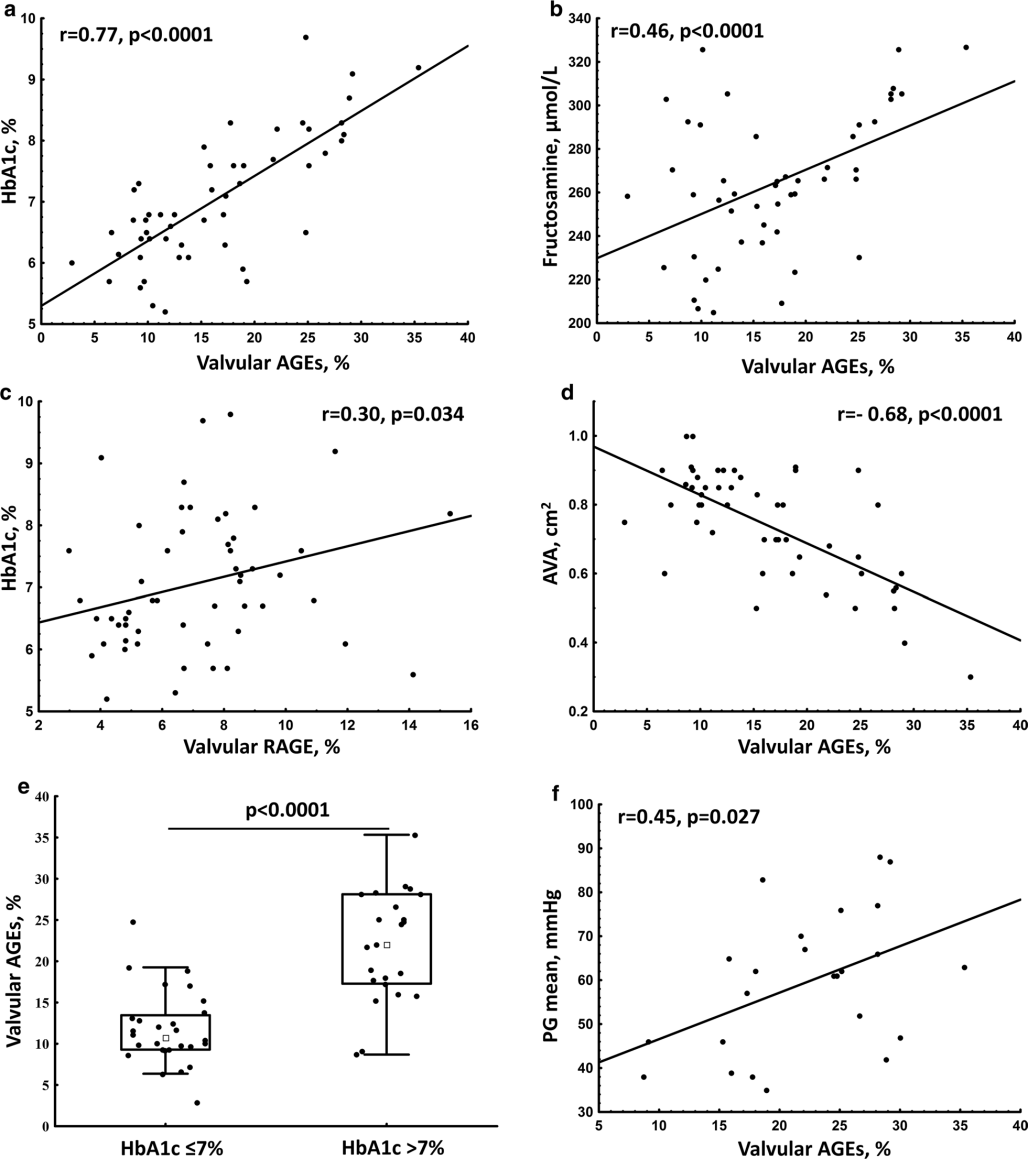
Associations of valvular AGEs and RAGE with glycation markers and echocardiographic parameters in patients with AS and concomitant DM. Associations of: **a** the expression of valvular advanced glycation end products (AGEs) with glycated hemoglobin (HbA1c), **b** valvular AGEs with serum fructosamine, **c** valvular AGEs receptor (RAGE) with HbA1c, **d** valvular AGEs with aortic valve area (AVA), **e** expression of valvular AGEs stratified according to elevated HbA1c levels, and **f** valvular AGEs with mean transvalvular pressure gradient (PG)

In DM patients the amount of valvular AGEs was positively associated with HbA1c and fructosamine levels (Fig. 2a, b). The valvular RAGE correlated positively with HbA1c (Fig. 2c) but not with plasma glucose, insulin or fructosamine levels (data not shown). In DM, but not in nonDM patients, we found negative association between the amount of valvular AGEs and aortic valve area (AVA; Fig. 2d), even after adjustment for potential confounders (Table 3). Similar association was not observed for valvular RAGE (Table 3). Furthermore, in DM patients with HbA1c > 7% (n = 24, 48%), we observed 1.2-fold higher expression of AGEs compared to those with HbA1c ≤ 7% (Table 2; Fig. 2e) which correlated with PG_mean_ (Fig. 2f).

**Table 3:** Multivariate associations between echocardiographic parameters and valvular or plasma AGEs and RAGE levels

Variable	AVA, estimate (95% CI)	p-value	Mean gradient, estimate (95% CI)	p-value	Maximum gradient, estimate (95% CI)	p-value
Valvular AGEs, %	− 0.29 (− 0.10; − 0.47)	0.0035	0.24 (0.05; 0.43)	0.014	0.34 (0.15; 0.52)	0.0005
Valvular RAGE, %	− 0.13 (− 0.32; 0.066)	0.19	0.082 (− 0.11; 0.28)	0.41	0.09 (− 0.11; 0.29)	0.36
Plasma AGEs, ng/mL	− 0.20 (− 0.0041; − 0.39)	0.045	0.13 (− 0.069; 0.32)	0.20	0.04 (− 0.15; 0.24)	0.66
Plasma sRAGE, pg/mL	− 0.26 (− 0.063; − 0.45)	0.01	0.13 (− 0.07; 0.33)	0.20	0.18 (− 0.16; 0.38)	0.071
Valvular AGEs/sRAGE ratio	− 0.19 (0.007; − 0.39)	0.058	0.18 (− 0.02; 0.38)	0.078	0.19 (− 0.008; 0.39)	0.059

All models were adjusted for age, hypertension, hypercholesterolemia, the use of statin, and angiotensin-converting enzyme inhibitors. *CI* confidence interval; for other abbreviations see Tables 1 and 2

### Plasma levels of AGEs and sRAGE

**Fig. 3:**
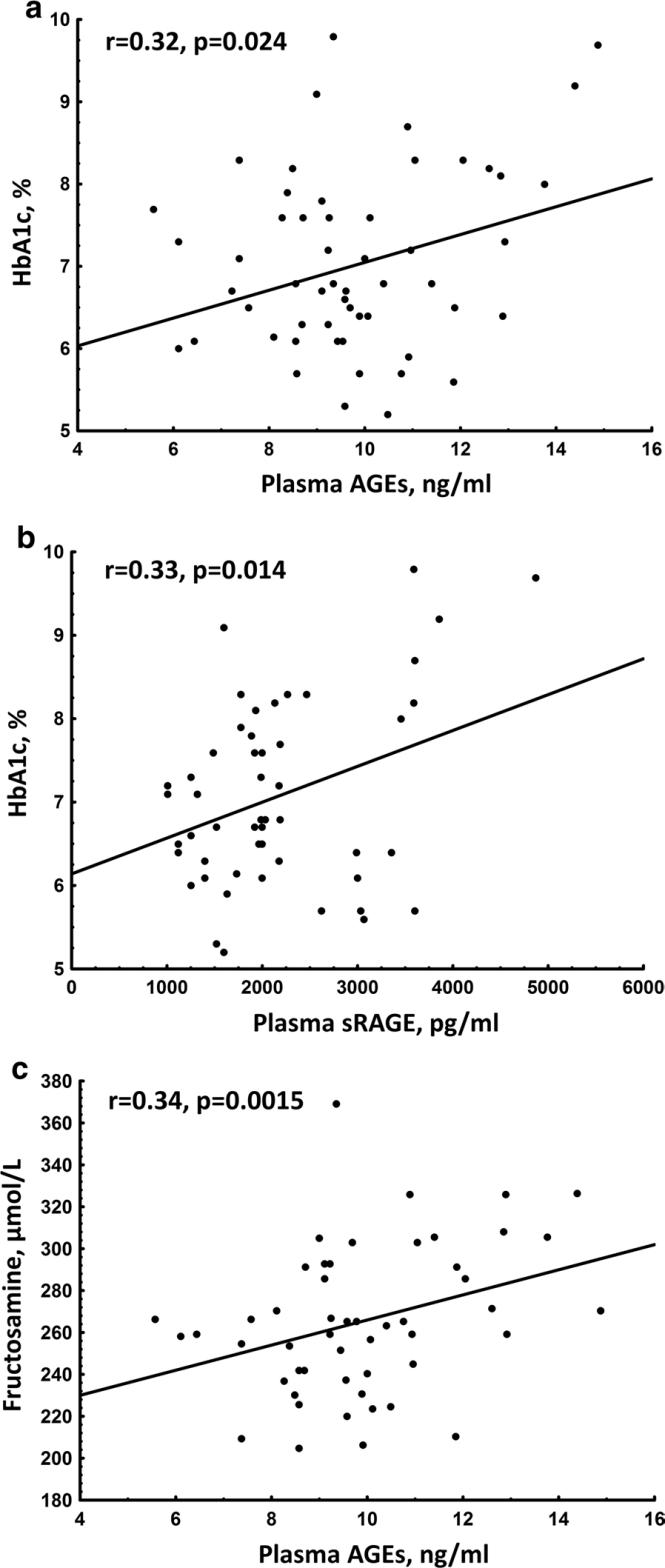
Associations of plasma AGEs and RAGE with glycation markers in patients with AS and concomitant DM. Associations of: **a** plasma levels of advanced glycation end products (AGEs) with glycated hemoglobin (HbA1c), **b** plasma levels of AGE receptor (RAGE) with HbA1c, **c** plasma AGEs with serum fructosamine levels

DM compared to nonDM patients had 12-fold higher plasma concentrations of AGEs (9.55 [8.56–10.92] vs. 0.73 [0.68–0.77] ng/mL; p < 0.0001) and 1.3-fold higher plasma sRAGE levels (1982 [1517–2613] vs. 858 [648–971] pg/mL; p < 0.0001). Of note, in the DM group plasma AGEs correlated with sRAGE levels (r = 0.61, p < 0.0001) and plasma concentrations of AGEs and sRAGE were positively associated with HbA1c levels (Fig. 3a, b). Importantly, in the DM group, plasma AGEs levels positively correlated with fructosamine (Fig. 3c). In DM, but not in nonDM patients, HbA1c was associated with fructosamine levels (r = 0.54, p < 0.0001). Associations of plasma AGEs and sRAGE levels with HbA1c or fructosamine were not observed in the nonDM group (data not shown).

Moreover, in the DM group, plasma levels of AGEs correlated positively with valvular AGEs expression (r = 0.67, p = 0.037) but valvular RAGE was not associated with sRAGE (p > 0.05).

**Fig. 4:**
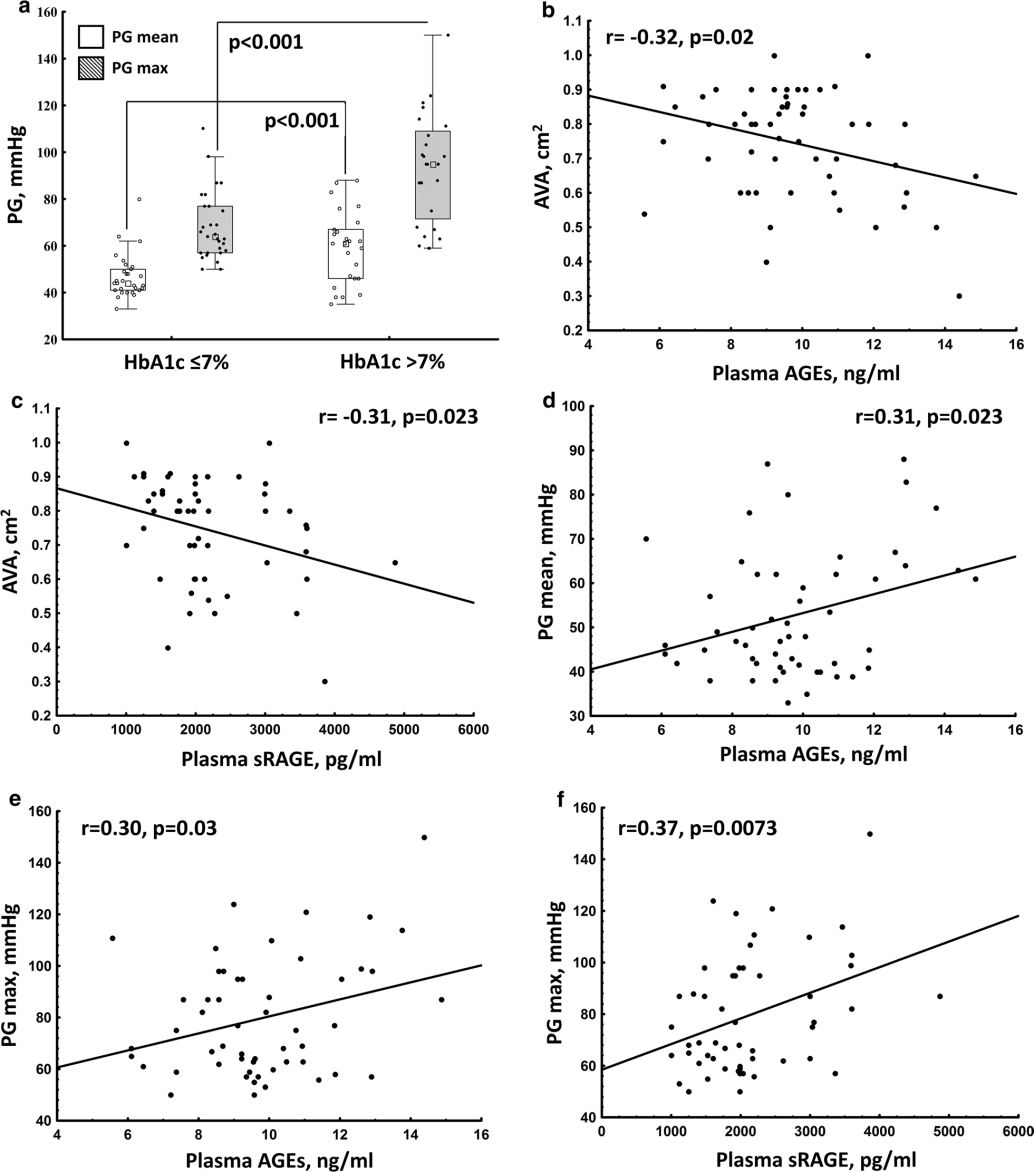
Associations of plasma AGEs and RAGE with echocardiographic parameters in patients with AS and concomitant DM. Associations of: **a** mean and maximal transvalvular pressure gradients (PG) stratified according to elevated levels of glycated hemoglobin (HbA1c), **b** plasma levels of advanced glycation end products (AGEs) with aortic valve area (AVA), **c** plasma levels of AGEs receptor (RAGE) with AVA, **d** plasma levels of AGEs with mean PG or **e** maximal PG, and **f** plasma RAGE levels with maximal PG

There were no associations between echo parameters and HbA1c or glucose levels in the whole group of DM subjects. However, DM patients with HbA1c > 7.0% compared to those with HbA1c ≤ 7.0% were characterized by higher PG_max_ and PG_mean_ (Table 2; Fig. 4a).

In DM patients plasma levels of AGEs and sRAGE were associated with the disease severity reflected by AVA (Fig. 4b, c), even after adjustment for defined confounders (Table 3). Moreover, plasma AGEs (but not sRAGE) levels correlated positively with PG_mean_ (Fig. 4d), while PG_max_ was associated with both AGE and sRAGE plasma levels (Fig. 4e, f). However, associations of transvalvular gradients with plasma AGEs and sRAGE were no more significant after adjustment for defined confounders (Table 3).

### Ratio of valvular AGEs and plasma sRAGE levels (AGEs/sRAGE)

We found significant associations between the AGEs/sRAGE ratio and glucose levels (r = 0.36, p = 0.077), HbA1c (r = 0.46, p = 0.0004) as well as with echocardiographic parameters such as AVA (r = − 0.41, p = 0.0033), PG_mean_ (r = 0.32, p = 0.019), PG_max_ (r = 0.37, p = 0.007). However, after adjustment for defined confounders all estimates showed only a tendency to be significant (Table 3).

## Discussion

To the best of our knowledge, this study is the first to show that: (1) patients with AS and concomitant DM compared to nonDM patients with tricuspid aortic valves, demonstrate abundant valvular accumulation of AGEs and RAGE associated with AS severity. Interestingly, poorly controlled DM was associated with the highest valvular AGEs expression; (2) the expression of valvular AGEs corresponded to AGEs level in plasma but this association held no true for valvular RAGE and sRAGE, and (3) plasma levels of AGEs and sRAGE were correlated with AVA but not with transvalvular pressure gradients.

### AGEs involvement in AS

Our results showing that enhanced valvular expression of AGEs associated with AS severity in AS patients with concomitant DM are in line with studies performed in mice, in which AGEs accumulation resulted in aortic valve degeneration [16]. Previously, we have shown that diabetes is associated with increased valvular inflammation, measured by valvular CRP expression [23]. The present study provides the initial evidence for a proinflammatory effect of valvular AGEs accumulation. Despite the fact that AGEs are accumulated as a result of hyperglycemia, their effects can occur independently of glycemic control and their levels better predicted both DM progression and vascular calcification than HbA1c [24]. Although glycemic control is important, it is not sufficient to prevent complications of diabetes. Other factors, such as oxidative stress, may be more important mediators of advanced glycation than hyperglycemia per se in patients already receiving interventions directed to improve glycemic control [6]. On the other hand, our study provided additional data that both biomarkers of long-term glycemic control (HbA1c and fructosamine) show associations with plasma and valvular AGEs. Arguably, they may give insight into disease course in AS patients with concomitant DM, especially in those with mild-to-moderate AS. However, further studies are needed to explore this issue.

It is known that AGEs accumulation in the extracellular matrix of vessels leads to structural and functional changes of collagen and subsequent cardiovascular complications [22]. Therefore, it might be hypothesized that more severe AS observed in patients with concomitant DM is a result of AGE-related valvular collagen cross-linking leading to enhanced inflammation, oxidative stress, and calcification of the leaflets. Studying a cohort with confirmed normal-flow high-gradient AS, we observed an association between valvular AGEs expression and AVA in DM. This observation was further corroborated by correlation of valvular AGEs expression and mean transvalvular pressure gradient. Considered the fact that also plasma AGEs levels were associated with AVA, it becomes evident that AGEs have multilevel interrelation with AS severity in diabetics, even at the very late stage of the disease, where surgical intervention is inevitable.

Our novel observation is that poorly controlled DM was associated with the highest valvular AGEs expression, which correlated not only with AVA but also with transvalvular pressure gradients. The influence of diabetes on AS progression has been previously studied. Katz et al. [7] have shown that the metabolic syndrome and DM are associated with increased risk of valvular calcification, and AS prevalence is associated with increasing number of metabolic syndrome components. Kamalesh et al. [25] have shown an influence of diabetes on AS progression in patients with mild AS, but not in those with heavily calcified stenotic valves. They speculated that once the valve is calcified, the diabetes-driven inflammation may play less of a role in AS progression than in a valve with mild lesions. Recently, Larsson et al. in a prospective cohort study comprising > 70,000 individuals have confirmed that type 2 DM is associated with an increased risk of AS (hazard ratio: 1.34; 95% confidence interval 1.05–1.71) [26]. On the contrary, Testuz et al. [27] failed to demonstrate an association between AS progression and metabolic syndrome or diabetes during 3-years follow-up. However, the authors evaluated only levels of fasting glucose, reflecting short-term glucose dynamics. Neither the levels of HbA1c, fructosamine nor AGEs in their cohort were assessed [27], while our study showed that in AS patients with well-controlled type 2 DM an influence of hyperglycemia on AS severity is minor.

### RAGE involvement in AS

Despite the role of RAGE in vascular calcification has been previously demonstrated in animal models [28, 29], our study showed that RAGE in contrast to AGEs is poorly associated with AS severity measures. Moreover, we found no associations between AGE/sRAGE ratio and AS severity after adjustment for potential confounders, while this ratio has been suggested as a more sensitive marker than single factors [22, 30]. These discrepancies may be connected with the fact that there are different species of RAGE, including the membrane RAGE, responsible for the harmful effects of AGEs and circulating RAGE isoforms which are protective against AGEs due to competing with the tissue RAGE for binding of AGEs [31]. Thus, this dual nature of RAGE, together with an increased consumption of AGE by sRAGE in subjects with impaired glucose metabolism [32] may disturb a direct associations between RAGE and parameters describing AS severity. Moreover, we showed relatively high levels of sRAGE observed in DM compared to nonDM patients, while there are studies reporting decreased, increased or unchanged sRAGE levels in DM patients compared to subjects without DM [33–35]. This difference may be explained by a presence of several confounding factors, with major impact of DM duration. It has been shown that the longer duration of DM the higher AGEs generation and AGE-stimulated increased RAGE expression [36]. Moreover, the presence of hypertension, use of antihypertensive drugs or inflammatory diseases can affect plasma sRAGE levels [36].

### Study limitations

This study has several limitations. The number of study participants was limited, however, the study was adequately powered to detect intergroup differences in AGEs levels. Moreover, almost half of the DM patients had well-controlled diabetes with HbA1c < 7%. This could be a reason that we failed to show associations of valvular AGEs and RAGE accumulation with valvular inflammation or oxidation. On the other hand, more sensitive markers of inflammation and oxidation, or using another type of antibodies for immunostaining, could be more informative. Second, although the analysis of valvular expression of AGEs and RAGE along with IL-6 and ROS was semiquantitative, but assessed by two independent investigators, results of such analyses should be interpreted with caution. Third, metformin has the ability to reduce AGEs accumulation [37], and it should be mentioned that most of DM patients in our cohort received this drug. AGEs levels in newly diagnosed or untreated DM patients might be higher.

## Conclusions

We conclude that AGEs and RAGE accumulate within stenotic aortic valves in DM patients, and the degree of this accumulation is associated with AS severity. Moreover, we found that plasma AGE and sRAGE levels were associated with AVA, thus they may be considered as new biomarkers of the AS course in patients with concomitant type 2 DM. Further studies are urgently needed to elucidate whether more strict control of diabetes is capable of slowing AS progression in patients with mild-to-moderate valvular disease.

## Data Availability

The datasets used and/or analyzed during the current study are available from the corresponding author on reasonable request.

## Abbreviations

AGEs: Advanced glycation end products
AS: Aortic stenosis
DM: Aortic stenosis with concomitant type 2 diabetes
AVA: Aortic valve area
HbA1c: Glycated hemoglobin
IL-6: Interleukin 6
PG_mean/max_: Transvalvular pressure gradients mean or maximal
RAGE: AGEs receptor
ROS: Reactive oxygen species

## References

[1] Lindroos M, Kupari M, Heikkilä J, Tilvis R (1993). Prevalence of aortic valve abnormalities in the elderly: an echocardiographic study of a random population sample. J Am Coll Cardiol.

[2] Miller JD, Weiss RM, Heistad DD (2011). Calcific aortic valve stenosis: methods, models, and mechanisms. Circ Res.

[3] Ngo MV, Gottdiener JS, Fletcher RD, Fernicola DJ, Gersh BJ (2001). Smoking and obesity are associated with the progression of aortic stenosis. Am J Geriatr Cardiol..

[4] Czestkowska E, Rożanowska A, Długosz D, Bolt K, Świerszcz J, Kruszelnicka O (2019). Depressed systemic arterial compliance and impaired left ventricular midwall performance in aortic stenosis with concomitant type 2 diabetes: a retrospective cross-sectional study. Cardiovasc Diabetol..

[5] Giacco F, Brownlee M (2010). Oxidative stress and diabetic complications. Circ Res.

[6] Forbes JM, Soldatos G, Thomas MC (2005). Below the radar: advanced glycation end products that detour “around the side”. Is HbA1c not an accurate enough predictor of long term progression and glycaemic control in diabetes?. Clin Biochem Rev..

[7] Katz R, Wong ND, Kronmal R, Takasu J, Shavelle DM, Probstfield JL (2006). Features of the metabolic syndrome and diabetes mellitus as predictors of aortic valve calcification in the Multi-Ethnic Study of Atherosclerosis. Circulation.

[8] Hegab Z, Gibbons S, Neyses L, Mamas MA (2012). Role of advanced glycation end products in cardiovascular disease. World J Cardiol..

[9] Kizer JR, Benkeser D, Arnold AM, Ix JH, Mukamal KJ, Djousse L (2014). Advanced glycation/glycoxidation endproduct carboxymethyl-lysine and incidence of coronary heart disease and stroke in older adults. Atherosclerosis..

[10] Kiuchi K, Nejima J, Takano T, Ohta M, Hashimoto H (2001). Increased serum concentrations of advanced glycation end products: a marker of coronary artery disease activity in type 2 diabetic patients. Heart.

[11] Di Pino A, Currenti W, Urbano F, Scicali R, Piro S, Purrello F (2017). High intake of dietary advanced glycation end-products is associated with increased arterial stiffness and inflammation in subjects with type 2 diabetes. Nutr Metab Cardiovasc Dis..

[12] Thorpe SR, Baynes JW (2003). Maillard reaction products in tissue proteins: new products and new perspectives. Amino Acids.

[13] Francis-Sedlak ME, Moya ML, Huang JJ, Lucas SA, Chandrasekharan N, Larson JC (2010). Collagen glycation alters neovascularization in vitro and in vivo. Microvasc Res.

[14] Wautier MP, Chappey O, Corda S, Stern DM, Schmidt AM, Wautier JL (2001). Activation of NADPH oxidase by AGE links oxidant stress to altered gene expression via RAGE. Am J Physiol Endocrinol Metab.

[15] Branchetti E, Bavaria JE, Grau JB, Shaw RE, Poggio P, Lai EK (2014). Circulating soluble receptor for advanced glycation end product identifies patients with bicuspid aortic valve and associated aortopathies. Arterioscler Thromb Vasc Biol.

[16] Hofmann B, Yakobus Y, Indrasari M, Nass N, Santos AN, Kraus FB (2014). RAGE influences the development of aortic valve stenosis in mice on a high fat diet. Exp Gerontol.

[17] Maresca AM, Guasti L, Bozzini S, Mongiardi C, Tandurella N, Corso R (2019). sRAGE and early signs of cardiac target organ damage in mild hypertensives. Cardiovasc Diabetol..

[18] Du R, Zhang RY, Lu L, Shen Y, Pu LJ, Zhu ZB (2018). Increased glycated albumin and decreased esRAGE levels in serum are related to negative coronary artery remodeling in patients with type 2 diabetes: an Intravascular ultrasound study. Cardiovasc Diabetol..

[19] Gelžinský J, Mayer O, Seidlerová J, Mateřánková M, Mareš Š, Kordíková V (2020). Soluble receptor for advanced glycation end-products independently influences individual age-dependent increase of arterial stiffness. Hypertens Res.

[20] American Diabetes Association (2014). Diagnosis and classification of diabetes mellitus. Diabetes Care.

[21] Natorska J, Marek G, Hlawaty M, Sadowski J, Tracz W, Undas A (2011). Fibrin presence within aortic valves in patients with aortic stenosis: association with in vivo thrombin generation and fibrin clot properties. Thromb Haemost.

[22] Mayer O, Gelžinský J, Seidlerová J (2020). The role of advanced glycation end products in vascular aging: which parameter is the most suitable as a biomarker?. J Hum Hypertens.

[23] Natorska J, Wypasek E, Grudzień G, Sobczyk D, Marek G, Filip G (2012). Does diabetes accelerate the progression of aortic stenosis through enhanced inflammatory response within aortic valves?. Inflammation..

[24] Writing Team for the Diabetes Control and Complications Trial/Epidemiology of Diabetes Interventions and Complications Research Group (2003). Sustained effect of intensive treatment of type 1 diabetes mellitus on development and progression of diabetic nephropathy: the Epidemiology of Diabetes Interventions and Complications (EDIC) study. JAMA.

[25] Kamalesh M, Ng C, El Masry H, Eckert G, Sawada S (2009). Does diabetes accelerate progression of calcific aortic stenosis?. Eur J Echocardiogr..

[26] Larsson SC, Wallin A, Håkansson N, Stackelberg O, Bäck M, Wolk A (2018). Type 1 and type 2 diabetes mellitus and incidence of seven cardiovascular diseases. Int J Cardiol.

[27] Testuz A, Nguyen V, Mathieu T, Kerneis C, Arangalage D, Kubota N (2017). Influence of metabolic syndrome and diabetes on progression of calcific aortic valve stenosis. Int J Cardiol.

[28] Li F, Cai Z, Chen F, Shi X, Zhang Q, Chen S (2012). Pioglitazone attenuates progression of aortic valve calcification via down-regulating receptor for advanced glycation end products. Basic Res Cardiol.

[29] Wang B, Cai Z, Liu B, Liu Z, Zhou X, Dong N (2017). RAGE deficiency alleviates aortic valve calcification in ApoE−/− mice via the inhibition of endoplasmic reticulum stress. Biochim Biophys Acta Mol Basis Dis..

[30] Prasad K (2014). Low levels of serum soluble receptors for advanced glycation end products, biomarkers for disease state: myth or reality. Int J Angiol.

[31] Grauen Larsen H, Marinkovic G, Nilsson PM, Nilsson J, Engstrom G, Melander O (2019). High plasma sRAGE (soluble receptor for advanced glycation end products) is associated with slower carotid intima-media thickness progression and lower risk for first-time coronary events and mortality. Arterioscler Thromb Vasc Biol.

[32] Miranda ER, Somal VS, Mey JT, Blackburn BK, Wang E, Farabi S (2017). Circulating soluble RAGE isoforms are attenuated in obese, impaired-glucose-tolerant individuals and are associated with the development of type 2 diabetes. Am J Physiol Endocrinol Metab.

[33] Devangelio E, Santilli F, Formoso G, Ferroni P, Bucciarelli L, Michetti N (2007). Soluble RAGE in type 2 diabetes: association with oxidative stress. Free Radic Biol Med..

[34] Nakamura K, Yamagishi S, Adachi H, Kurita-Nakamura Y, Matsui T, Yoshida T (2007). Elevation of soluble form of receptor for advanced glycation end products (sRAGE) in diabetic subjects with coronary artery disease. Diabetes Metab Res Rev..

[35] Grossin N, Wautier MP, Meas T, Guillausseau PJ, Massin P, Wautier JL (2008). Severity of diabetic microvascular complications is associated with a low soluble RAGE level. Diabetes Metab..

[36] Yamagishi S, Matsui T (2010). Soluble form of a receptor for advanced glycation end products (sRAGE) as a biomarker. Front Biosci.

[37] Ahmad S, Shahab U, Baig MH, Khan MS, Khan MS, Srivastava AK (2013). Inhibitory effect of metformin and pyridoxamine in the formation of early, intermediate and advanced glycation end-products. PLoS ONE.

